# Characterization and practical use of self-compatibility in outcrossing grass species

**DOI:** 10.1093/aob/mcab043

**Published:** 2021-03-23

**Authors:** Claudio Cropano, Iain Place, Chloé Manzanares, Javier Do Canto, Thomas Lübberstedt, Bruno Studer, Daniel Thorogood

**Affiliations:** 1 Molecular Plant Breeding, Institute of Agricultural Sciences, ETH Zurich, Zurich, Switzerland; 2 Deutsche Saatveredelung AG, Lippstadt, Germany; 3 Institute of Biological, Environmental and Rural Sciences, Aberystwyth University, Aberystwyth, UK; 4 Instituto Nacional de Investigación Agropecuaria (INIA), 4500 Tacuarembó, Uruguay; 5 Department of Agronomy, Iowa State University, Ames, IA, USA

**Keywords:** Self-compatibility, self-incompatibility, inbreeding, Poaceae, introgression, inbreeding depression, heterosis, *F*_1_ hybrid breeding

## Abstract

**Background:**

Self-incompatibility (SI) systems prevent self-fertilization in several species of Poaceae, many of which are economically important forage, bioenergy and turf grasses. Self-incompatibility ensures cross-pollination and genetic diversity but restricts the ability to fix useful genetic variation. In most inbred crops it is possible to develop high-performing homozygous parental lines by self-pollination, which then enables the creation of *F*_1_ hybrid varieties with higher performance, a phenomenon known as heterosis. The inability to fully exploit heterosis in outcrossing grasses is partially responsible for lower levels of improvement in breeding programmes compared with inbred crops. However, SI can be overcome in forage grasses to create self-compatible populations. This is generating interest in understanding the genetical basis of self-compatibility (SC), its significance for reproductive strategies and its exploitation for crop improvement, especially in the context of *F*_1_ hybrid breeding.

**Scope:**

We review the literature on SI and SC in outcrossing grass species. We review the currently available genomic tools and approaches used to discover and characterize novel SC sources. We discuss opportunities barely explored for outcrossing grasses that SC facilitates. Specifically, we discuss strategies for wide SC introgression in the context of the *Lolium*–*Festuca* complex and the use of SC to develop immortalized mapping populations for the dissection of a wide range of agronomically important traits. The germplasm available is a valuable practical resource and will aid understanding the basis of inbreeding depression and hybrid vigour in key temperate forage grass species.

**Conclusions:**

A better understanding of the genetic control of additional SC loci offers new insight into SI systems, their evolutionary origins and their reproductive significance. Heterozygous outcrossing grass species that can be readily selfed facilitate studies of heterosis. Moreover, SC introduction into a range of grass species will enable heterosis to be exploited in innovative ways in genetic improvement programmes.

## Introduction

Self-incompatibility (SI) is the most widespread mechanism for promoting outcrossing in hermaphrodite flowering plants. Described by Charles Darwin as ‘the ability of some plants to reject their own pollen’, SI is considered the mating system of ancestral angiosperms (Allen and Hiscock, 2008). The first flowering plants retained SI to maximize cross-pollination and genetic diversity to adapt to and expand into a wide range of habitats. Self-incompatibility systems in angiosperms are, in most studied cases, controlled by one highly polymorphic *S* locus containing at least two tightly linked genes, encoding the male and female-specific SI determinants that mediate the self/non-self-pollen recognition. If the *S* alleles carried by the pollen and pistil match, the pollen is recognized as ‘self’, thus preventing self-fertilization and the resulting inbreeding.

The breakdown of SI caused a change from outcrossing to self-fertilization in the evolutionary history of many flowering plants (Vekemans *et al.*, 2014). During angiosperm evolution and expansion, SI has frequently been lost irreversibly through loss-of-function mutations (Igic *et al.*, 2008). Self-compatibility (SC) evolved as a reproduction strategy in plants as, among other factors, a preadaptation to plant domestication (Iriondo *et al.*, 2018). As a consequence of the high level of inbreeding resulting from self-fertilization, deleterious recessive mutations were revealed that early farmers selected against to propagate plants with favourable alleles contributing to useful traits (Abbo *et al.*, 2012; Kantar *et al.*, 2017).

Understanding how SC has arisen is fundamental for plant evolution, population dynamics and plant domestication studies. In practice, SC has enabled the production of superior inbred lines purged of deleterious recessive alleles. Homozygous lines from divergent backgrounds can be crossed to create *F*_1_ hybrids with increased performance, a phenomenon known as heterosis. Understanding of this phenomenon has been enhanced by the development of mapping populations consisting of homozygous lines in which combinations of alleles from the crossed parents are fixed. These lines can be propagated indefinitely as seeds.

Thus, genetic mapping and dissection of agronomic traits have been useful strategies for the development of targeted approaches to DNA marker trait selection in many inbred crops by developing durable immortalized inbred line-based genetic resources. These include recombinant inbred lines (RILs) produced through recurrent rounds of inbreeding (Burr *et al.*, 1988) and near-isogenic lines (NILs) produced by repeated backcrosses of an RIL to another population and selfing to produce an overall homogeneous population varying in the presence of introgressed genomic segments from the initial RIL in proximity to a particular target region (Kaeppler *et al.*, 1993). Furthermore, homozygous lines can be produced rapidly and with a substantially smaller population by using doubled haploid techniques (Guzy-Wrobelska and Szarejko, 2003). These populations cannot be produced in outcrossing species, and in grasses, such as perennial ryegrass (*Lolium perenne*), genetic mapping studies employ highly heterozygous pseudo-*F*_1_ or pseudo-*F*_2_ populations that need to be maintained clonally. This is labour-intensive and potentially compromises the genetic integrity of the population over time.

Several self-incompatible species of the Poaceae are economically valuable forage crops and represent a fundamental component in many ruminant production systems. Self-incompatibility has acted as a barrier to their improvement and forage yield advances in breeding have been small (Woodfield, 1999; Wilkins and Humphreys, 2003). Currently, because of the breeding behaviour and the self-incompatible nature of the plants, forage crop breeding is predominantly based on lengthy population improvement programmes using recurrent selection, achieving modest genetic gains compared to self-compatible crop species (Laidig *et al.*, 2014; McDonagh *et al.*, 2014). This might be because in outcrossing species it is extremely challenging to produce the inbred lines necessary to fully exploit heterosis. Instead, breeding activity has focused on recurrent selection, accumulating and fixing favourable genes with additive effects on desired traits in ‘synthetic’ population varieties. In such populations, although heterosis will occur at a proportion of heterozygous loci, these will continue to segregate through the breeding process despite the imposed selection. Perhaps more significantly, the maintenance of high levels of heterosis is even less likely during the process of seed multiplication, where there is no agronomic selection.

The need to accelerate breeding progress in forage grasses for traits such as biomass and seed yield, nutritional quality and disease resistance is stimulating grass breeders to develop hybrid breeding technologies as an alternative to recurrent population selection strategies. For this reason, overcoming SI and exploiting SC to breed forage grasses as an inbred line-based crop is gaining interest (Do Canto *et al.*, 2016; Herridge *et al.*, 2019).

With SC made available in inherently highly heterozygous self-incompatible plant populations, there are opportunities to discover a potentially greater pool of novel genomic regions and underlying candidate genes associated with heterotic responses than would be found in less genetically diverse inbred crop species. Such associations can then be practically exploited in the same way that variation in immortalized populations has enabled the genetic dissection of various traits in self-compatible species and a genomic marker-assisted approach to *F*_1_ hybrid plant breeding in several inbred crops (Xu *et al.*, 2017).

Several SC sources in predominantly outcrossing and agronomically relevant grasses have been reported so far. These arise mainly from spontaneous mutations at the *S* and/or *Z* loci that disrupt the initial self-/non-self-recognition between pollen and stigma. Mutations in so-called SI modifier genes can also lead to SC, by interrupting the downstream cascade triggered by the initial self-recognition response (Do Canto *et al.* 2016). However, a detailed molecular understanding of how SC arises, along with its systematic manipulation and introgression in a wide germplasm resource, is still limiting progress.

Here, we review the current knowledge of SI and SC in grasses and we discuss how recent developments in genomics and DNA sequencing technologies will help characterize novel SC sources in grasses. To conclude, we explore possibilities, so far considered impractical, that SC facilitates in the context of grass improvement programmes. In this final section we focus on how using SC in forage grasses will enable a greater fundamental understanding of heterosis and an ability to manipulate it in novel ways to fully exploit the genetic diversity potentially offered.

## SELF-INCOMPATIBILITY IN THE GRASSES

Genetic mechanisms of SI in the angiosperms are phylogenetically diverse and often family-specific, indicating several *de novo* evolutionary pathways (Bateman, 1952). Physiological SI mechanisms can be classified as gametophytic or sporophytic, but the specific mechanisms, as well as the number of genetic determinants involved, vary between families. Identification of SI-determinant genes has only been achieved for SI systems controlled by a single locus, called *S*, such as those of the Solanaceae, Rosaceae, Plantaginaceae, Brassicaceae and Papaveraceae, discussed in detail in a recent review (Muñoz-Sanz *et al.*, 2020). Despite the involvement of different molecular actors, the underlying genetic principles of all single-locus SI systems are similar. The *S* locus consists of at least two tightly linked genes that together form a non-recombining *S* haplotype: one gene encodes the female determinant of SI, which is expressed in the pistil, and another gene encodes the male determinant of SI, which is expressed in the pollen (gametophytically controlled SI) or in tapetal cells (sporophytically controlled SI). In a self-pollination event, the products of male and female determinants interact with each other and trigger the pollen inhibition response that leads to SI (Brennan *et al.*, 2011).

In comparison with single-locus SI systems, the SI systems under the control of more than one locus are considerably more challenging to study, the additional SI loci adding a level of complexity to the understanding of pollen–stigma interactions. Known multi-locus SI systems are controlled by two loci in Poaceae (Hayman, 1956; Lundqvist, 1956), three loci in buttercup (*Ranunculus* spp.) (Lundqvist *et al.*, 1973; Lundqvist, 1990) and four loci in common beet (*Beta vulgaris*) (Lundqvist *et al.*, 1973) and martagon lily (*Lilium martagon*) (Lundqvist, 1991). Of these, the SI system of the grass family is the best documented.

The gametophytic SI system of the Poaceae is controlled by two independent loci, designated *S* and *Z*, which are located on chromosomes 1 and 2, respectively (Thorogood *et al.*, 2002). Self-fertilization is prevented when identical *S* and *Z* haplotypes are shared by both pollen and stigma. This two-locus mechanism was first reported over 60 years ago in sunolgrass (*Phalaris coerulescens*) (Hayman, 1956) and rye (*Secale cereale*) (Lundqvist, 1956), later being confirmed in *L. perenne* (Cornish *et al.*, 1979). Despite several efforts to identify the SI-determinant genes, their identity and function are yet to be fully elucidated. However, considerable progress has been made in recent years. Through a combination of fine mapping, genome sequencing, transcriptome analysis and comparative sequence analysis, Manzanares *et al.* (2016*a*) identified and evaluated potential candidate genes for the *L. perenne* pollen *S*-locus component. Three candidate genes segregated with the *S* locus and were also overexpressed in reproductive tissues, with two upregulated in stigma tissue and a single gene upregulated in pollen. The gene encoding a protein (LpSDUF247) containing the DUF247 domain of unknown function appeared to be the most likely candidate for the pollen SI determinant as it possessed the high level of allelic variability expected of an SI determinant gene affected by frequency-dependent selection (Fearon *et al.*, 1994). LpSDUF247 was predicted to have a C-terminal transmembrane helix and an extracellular domain, suggesting that it may function as a ligand on the pollen surface (Manzanares *et al.*, 2016*a*). The variability between alleles was not evenly distributed across the protein sequence, with the region from the transmembrane domain to the C-terminus being more conserved. In addition, all self-compatible grass species for which *LpSDUF247* orthologue sequence information was available possessed either premature stop codon mutations or large deletions in the predicted protein sequence (Manzanares *et al.*, 2016*a*). However, no genes were proposed as a candidate for the stigma *S* determinant, possibly because a gap in the derived sequence of the *S* locus identified might have prevented its identification.

Expression profiling and allelic diversity assessment were also employed by Shinozuka *et al.* (2010) in an attempt to identify candidate genes for the *L. perenne Z* locus. Two genes were identified as plausible candidates for either the pollen or stigma determinants. Interestingly, one of these was a gene encoding a protein containing a *DUF247* domain (*LpDUF247*) similar to the *S* locus-linked *LpSDUF247* gene, with the other candidate gene encoding either a putative tetratricopeptide repeat-like domain-containing protein or a ubiquitin-specific protease (LpTC116908). These findings lend support to a hypothesis put forward by Lundqvist (1962) that the two-locus SI system in grasses originated from a duplication of an initial single SI locus. Orthologues of *LpSDUF247* have also been identified in other grass species, such as wild rice (*Oryza barthii*) and sorghum (*Sorghum bicolor*), suggesting that this gene could function as an SI determinant throughout the Poaceae (Manzanares *et al.*, 2016*a*). Although the candidate genes for the grass SI determinants here discussed have not been functionally characterized, preliminary studies found that calcium (Ca^2+^) signalling and protein phosphorylation are involved in the recognition and/or inhibition of incompatible pollen. Two independent expression studies found that transcripts predicted to code for proteins containing calcium-binding domains were enriched during the SI response in *L. perenne* and in sheepgrass (*Leymus chinensis*) (Yang *et al.*, 2009; Chen *et al.*, 2019). Moreover, Klaas *et al.* (2011) supported this hypothesis by showing that SI in *L. perenne* can be partially overcome by treating self-pollinated stigmas with chemical reagents known to affect Ca^2+^ channelling across membranes.

Map-based cloning and large-scale comparative genomics enabled by long-read assembly methods in combination with more targeted editing approaches will allow functional validation and confirmation of the candidate genes discussed here and provide useful insights into their molecular function in determining the SI response.

## SELF-COMPATIBILITY IN THE GRASSES

A significant portion of the literature regarding the genetic causes of SC is based on evolutionary studies aiming at unravelling the origins and dynamics of the transition of outbreeding species from SI to selfing. These studies, summarized in Supplementary Data Table S1 and referenced in Supplementary Data References S1, provide information on the genetic causes of SC in known sporophytic and gametophytic SI systems. Overall, these studies demonstrate that the causes of SC can be classified into two categories. Firstly, SC can arise from inactivation and/or loss-of-function mutations at SI determinants. Secondly, there are numerous examples of SC arising from genes unlinked to the SI-determinant genes, but that can affect their expression or mediate the up- or downstream pathways involved in the SI response.

Although self-incompatible species are predominant among the Poaceae, there are a number of naturally self-compatible species (for an exhaustive list see Do Canto *et al.*, 2016). Indications of how SC evolved in these species come from studies on darnel (*Lolium temulentum*), a naturally self-compatible species. By analysing SC segregation in backcrossing generations of *L. temulentum–L. perenne* and *L. temulentum*–annual ryegrass (*Lolium multiflorum*) interspecific hybrids, Thorogood and Hayward (1992) proposed that SC was under the control of a single locus and was caused by a mutation at the *Z* locus. This was inferred from distorted segregation of the *GOT*/*3* isozyme locus in selfed progeny of first-generation backcross plants. Information available at the time showed that the *GOT*/*3* locus was not linked to the *S* locus and therefore distorted segregation was inferred to have been associated with the *Z* locus. Later, linkage mapping experiments located the *GOT*/*3* locus on linkage group (LG) 3 (Jones *et al*., 2002), unlinked to either *S* or *Z*. However, an epistatic relationship between the *S* locus and a region of LG 3 that caused distorted segregation of markers, including the *GOT*/*3* locus on LG 3 (Thorogood *et al.*, 2002), strongly implies that SC in *L. temulentum* was in fact associated with the *S* locus. Corroboratively, Manzanares *et al.* (2016*a*) provided evidence that the orthologue of the *S*-locus candidate gene *LpSDUF247* in *L. perenne* carried a frameshift mutation in *L. temulentum* that alters the last 24 amino acids of the C-terminus, which might fully explain the failure of the SI system in this species.

Low levels of self-fertility in individuals of self-incompatible grass species, manifest as the ability of individual plants to set small amounts of selfed seeds on panicles isolated in paper bags, were known almost a century ago (Jenkin, 1930; Beddows, 1931). Early genetic studies on the breakdown of SI in *S. cereale* speculated on the existence of several mutations at *S* and *Z* (Lundqvist, 1958, 1962, 1968). In these studies, SI specificity was lost in the pollen but retained in the pistils, indicating that the mutants were pollen-part mutations. Later on, genotypes carrying putative mutations at *S* and *Z* in which the recognition between pollen and stigma was disrupted were reported in *Lolium* species (Thorogood and Hayward, 1991), *S. cereale* (Fuong *et al.*, 1993; Voylokov *et al.*, 1998) and *P. coerulescens* (Hayman and Richter, 1992).

**Fig. 1.**
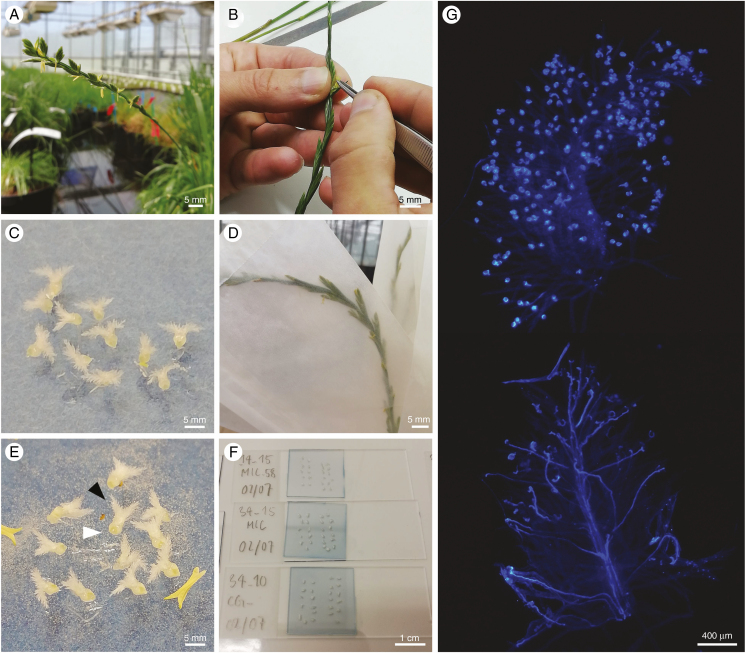
Phenotyping sc in grasses using an *in vitro* pollination assay. (A, b) virgin pistils are dissected from flowering heads and (c) placed on a petri dish with a medium containing agarose, sucrose and boric acid. (D) the heads are isolated in paper bags and, at the time of anthesis, they are shaken to release and collect fresh pollen. (E) the pollen is sprinkled homogeneously from the paper bags directly onto the virgin pistils. (F) at least 2 h after pollination, the stigmas (E, black arrow) are separated from the ovary (E, white arrow) and mounted on a microscope slide after staining with aniline blue solution, which stains selectively for callose visualized within pollen tube cell walls under UV light (Martin, 1959). (G) The level of pollen compatibility is assessed by observing the pollinated stigmas using UV fluorescence microscopy. In self-incompatible plants (above), self-pollen is usually bright, and a short and thickened pollen tube can be observed. In self-compatible plants (below) self-pollen grains are translucent and they can produce a bright pollen tube that grows through the stigma branches towards the style.

Another commonly reported route to SC in grasses is through loci unlinked to *S* and *Z*, as reported in *S. cereale* (Voylokov *et al.*, 1993; Egorova *et al.*, 2000) and *L. perenne* (Thorogood *et al.*, 2005). Genes underlying these loci likely have a function in the downstream cascade following the initial pollen–stigma recognition mediated by *S* and *Z* (Do Canto *et al.*, 2016). The most recent studies on non-*S* or non-*Z* sources of SC identified two genetically independent loci on LG 5 (Arias-Aguirre *et al.*, 2013; Do Canto *et al.*, 2018; Cropano *et al.*, 2021) and LG 6 (Slatter *et al.*, 2020) in two unrelated perennial ryegrass *F*_2_ populations segregating for SC. In both populations, using *in vitro* self-pollinations (Fig. 1), a similar 1:1 segregation in two phenotypic SC classes was observed: plants showing 50 % pollen compatibility, where half of the self-pollen germinated and grew a pollen tube upon contact with the stigma, and 100 % SC, where all self-pollen showed a compatible reaction. In both cases, it was concluded that SC was under the control of a single gametophytically acting pollen gene. Furthermore, Cropano *et al.* (2021) narrowed down the region to a 0.26-cM locus, allowing the identification of candidate genes that could be the target of functional characterization studies.

Disruption of SI can arise from non-genetic sources as well, including environmental factors such as temperature and humidity, leading to a condition referred to as pseudo-self-compatibility (Cornish *et al.*, 1980; Fearon *et al.*, 1983). The existence of genetic variation for pseudo-self-compatibility has been reported in *L. perenne* (Elgersma *et al.*, 1989; Wilkins and Thorogood, 1992), but studies on its genetic regulation are lacking.

## TOWARDS THE DISCOVERY AND GENE IDENTITY OF NOVEL SC SOURCES IN THE GENOMICS ERA

Identifying sources of SC is more effective with the advent of next-generation sequencing (NGS)-based technologies and reference genome assemblies of grass species (Byrne *et al.*, 2015; Velmurugan *et al.*, 2016; Shinozuka *et al.*, 2017, ; Knorst *et al.*, 2019; Copetti *et al.*, 2021). Cost-effective and high-throughput NGS-based genotyping technologies such as genotyping-by-sequencing and chip arrays have been used in outcrossing grasses in a wide range of studies (Blackmore *et al.*, 2015; Fè *et al.*, 2015; Thorogood *et al.*, 2017; Begheyn *et al.*, 2018; Velmurugan *et al.*, 2016, 2018; Cericola *et al.*, 2018; Guo *et al.*, 2018). These tools make more sophisticated approaches such as genome-wide association studies (GWAS) an attractive option for the identification of SC loci. Indeed, GWAS overcome some limitations of quantitative trait loci (QTL) mapping in biparental populations, such as restricted allelic diversity and low mapping resolution, as it can be employed on multiparental populations exhibiting reduced linkage disequilibrium through accumulated historical recombination events and dense genome-wide molecular marker maps (Korte and Farlow, 2013). Performing GWAS is a suitable approach for the screening of natural populations and cultivars developed by breeders and does not require the lengthy preparation of mapping populations with controlled pedigrees. GWAS can also simultaneously identify multiple loci responsible for SC variation and provide first insights into their relationship (Thorogood *et al.*, 2017). Self-compatibility loci unlinked to SI determinants reported so far in grasses produce a 1:1 segregation into two SC groups, as mentioned earlier. In such cases, sequencing pools of hundreds of individuals belonging to either of the two contrasting SC groups (bulk segregant analysis) might be more effective in locating the causal mutation based on differential allele frequencies. This approach led to the identification of a mutation conferring pollen-part SC in sweet cherry (*Prunus avium*) (Ono *et al.*, 2018) and several loci harbouring modifier genes in *Arabidopsis lyrata* (Mable *et al.*, 2017).

Identifying narrow genomic regions harbouring SC using association genetics studies relies on the effective phenotyping of large populations. Almost all studies on SC in grasses have used *in vitro* self-pollination to phenotype SC with high resolution and accuracy (Fig. 1). However, this technique is extremely time-consuming and is restricted by the short flowering time of the plants. A valid alternative to phenotyping assays is the analysis of segregation distortion. In plants with an SI system, the segregation of an SC gene in certain crosses can lead to segregation distortion of markers surrounding the SC causal gene. Thus, the identification of distorted regions in carefully designed mapping populations can be used to map their position in the genome. This was recently demonstrated by Slatter *et al.* (2020), who found that the SC QTL in *L. perenne* on LG 6 and LG 5 coincided with maximum marker segregation distortion. Mapping segregation distortion overcomes the dependency of *in vitro* pollinations on flowering and it allows the screening of larger populations to achieve higher mapping resolution.

Once forward genetic experiments have identified loci responsible for SC variation, the causal genes, often selected from a list of candidates at each locus, need to be characterized and functionally validated. One way to achieve this is by assessing the phenotype resulting from a disruptive mutation in a coding sequence. Among other methods, TILLING (targeting induced local lesions in genomes) offers a high-throughput screening of point mutations at genes of interest in populations mutagenized by chemical treatment (McCallum *et al.*, 2000). In major crop species such as wheat (*Triticum aestivum*) (Slade *et al.*, 2005), maize (*Zea may*s) (Till *et al.*, 2004), pea (*Pisum sativum*) (Dalmais *et al.*, 2008), tomato (*Solanum lycopersicum*) (Piron *et al.*, 2010), barley (*Hordeum vulgare*) (Gottwald *et al.*, 2009) and others (Boualem *et al.*, 2014), TILLING has been adopted to isolate mutants with agronomically valuable traits. A TILLING platform is available for perennial ryegrass and is currently being used for the validation of SI candidate genes identified through forward genetic screens (Manzanares *et al.*, 2016*b*). The use of TILLING is especially appropriate for validating the effect of mutations on monogenic traits, such as SC, because a mutation in a gene controlling a monogenic trait will yield a readily detectable phenotype, allowing easier confirmation of its function. In addition, recent advances in genome editing through CRISPR/Cas9 and its implementation in forage grasses (Zhang *et al.*, 2020) makes this tool an attractive alternative for achieving targeted gene inactivation of SC candidate genes to unequivocally determine their function.

## PRACTICAL APPLICATIONS OF SC IN FORAGE GRASSES

In this section of the review we focus on strategies for using SC in grasses to exploit genetic diversity not normally readily available in the outcrossing grass species. Although challenging to unravel and fix, outcrossing species offer wider sources of genetic variation than inbreeding species (Hamrick and Godt, 1996).

### *Inbred lines and* F_*1*_*hybrids for plant breeding*

As forage crops, species of the Poaceae family are economically important, providing feed for ~80 % of bovine milk production (Huyghe *et al.*, 2014) and 70 % of meat production (Wilkins and Humphreys, 2003) worldwide. According to figures from the European Seed Certification Agencies Association (ESCAA) on the area utilized for the production of certified forage seeds in the European Union in 2018, species of the Poaceae are the most important (229 838 ha), together with the Fabaceae (229 878 ha). In addition, they are sown as turf grasses in stadiums and golf courses, parks and domestic lawns, where they are used for sport and recreation.

Currently, forage grasses are mainly improved as populations and released as synthetic cultivars. Cultivar development begins with the evaluation of widely spaced plants for traits such as flowering time, plant habit, disease resistance, abiotic stress tolerance and nutritive value. Selected plants are then polycrossed to create a base population, which is highly heterozygous and heterogeneous. As some traits, notably biomass, cannot reliably be evaluated in individual spaced plants, synthetic populations are produced by crossing selected individuals to produce seed for plot trials that are more representative of grass pastures. Individual full- or half-sib family progeny seeds are collected from polycrossed plants and evaluated in plot trials. Only families from the best progeny plots and those that indicate the best performance when put together (combining ability) proceed to the next generation. Rounds of recurrent phenotypic selection are made to gradually increase performance and at the same time populations are tested for uniformity. When the desired levels of performance and uniformity are reached the population is then intermated in isolation from possible contaminant pollen from outside sources for several generations to produce commercial quantities of seed. Levels of genetic improvement in forage grass yield have been noticeably modest in comparison with other crops (Pembleton *et al.*, 2015). Recent estimates indicated an annual genetic gain for biomass yield of 0.45 % in *L. perenne* and from 0.27 to 0.37 % for *L. multiflorum*, amongst the lowest compared with other major crops (Laidig *et al.*, 2014; McDonagh *et al.*, 2014), including forage *Z. mays* (Taube *et al*., 2020), where hybrid production technologies have been applied. To redress this deficiency, genomic selection technologies, developed in animal breeding, are currently being applied to recurrent selection programmes of outbreeding temperate forage grasses, most recently by Danish (Esfandyari *et al.*, 2020), French/Belgian (Keep *et al.*, 2020), New Zealand (Arojju *et al.*, 2020), Australian (Jighly *et al.*, 2019) and UK (Grinberg *et al.*, 2016) research groups and breeding companies. Four reviews of the technology applied to outcrossing grasses have been published (Hayes *et al.*, 2013; Yabe *et al.*, 2013; Lin *et al.*, 2014; Talukder and Saha, 2017). Regardless of how successful genomic selection proves to be, improvement is still restricted largely to the additive component of genetic variation, thus limiting potential gains. We suggest that an additional approach to forage grass breeding programmes, whereby the reproductive system is altered to facilitate inbred line production and the creation of hybrids, is required to maximally exploit genetic variation. The primary constraint limiting genetic gain in grasses is the inability to develop hybrid breeding strategies that effectively exploit heterosis (Brummer *et al.*, 2009). To boost the heterotic effect, the parents should be genetically distant to maximze the heterotic effect, and highly homozygous to restore heterozygosity and homogeneity in the *F*_1_ hybrid. Since its first implementation in *Z. mays*, *F*_1_ hybrid breeding has revolutionized grain yield during the 20th century in a wide range of crops. The extraordinary performance of early corn *F*_1_ hybrid varieties compared with synthetic varieties has stimulated forage grass breeders to find ways to develop hybrid forages.

Although not yet used on a commercial scale, hybrid schemes based on the intercrossing of two synthetic populations have been proposed for *L. perenne* (Foster, 1971*b*), switchgrass (*Panicum virgatum*) (Martinez-Reyna and Vogel, 2008) and other forage crop species (Brummer, 1999). These are assumed to produce ‘chance’ hybrid populations, where up to half of the progeny derives from a direct hybridization of the two populations. More sophisticated strategies were also proposed based on selective restriction of SI diversity (England, 1974; Posselt, 1993; Pembleton *et al.*, 2015) and on within-population crossing using cytoplasmic male-sterile plants (Rouwendal *et al.*, 1992; McDermott *et al.*, 2008; Islam *et al.*, 2014; Vogt *et al.*, 2020). These are predicted to substantially increase the proportion of hybrid seed produced.

The systematic development and use of self-compatible germplasm expands the possible hybrid breeding strategies available for forage grasses. Specifically, it offers the opportunity to design inbred line-based *F*_1_ hybrid breeding strategies by producing elite homozygous parental lines capturing favourable genetic diversity. These lines can be crossed for testing heterotic combinations to maximize combining ability in *F*_1_ hybrids. Although the challenges are considerable, the transition from populations to inbred line-based *F*_1_ hybrid breeding is gaining traction (Begheyn *et al.*, 2016; Do Canto *et al.*, 2016; Herridge *et al.*, 2019) and provides a long-awaited route to increased rates of genetic gain.

A major concern regarding the creation of homozygous inbred lines in forage grasses is the reduced fitness due to repeated cycles of self-pollination, a phenomenon called ‘inbreeding depression’. Inbreeding depression is caused by the unmasking of recessive deleterious mutations through inbreeding, that have accumulated over generations in obligate outcrossers. In outcrossing individuals, their deleterious effects are buffered as they exist mostly in the heterozygous state (Charlesworth and Willis, 2009). One way to overcome inbreeding depression is by eliminating, or ‘purging’, recessive deleterious alleles. This can be achieved by recurrent self-pollination of plants showing high performance at each selfing generation, until fully homozygous plants are created with a similar fitness to the original, highly heterozygous parent. This is particularly important in perennial species, like many of the forage grasses, that regularly reproduce asexually, accumulating recessive deleterious somatic mutations (Zhang *et al.*, 2019).

In grasses, inbreeding depression is severe and dependent on the genotype and number of cycles of inbreeding (Do Canto *et al.*, 2016). Moreover, because of SI, purging deleterious alleles is challenging. Only few and heavily depressed inbred progenies can be obtained by forced self-pollination in self-incompatible plants or by exploiting environmental conditions that favour pseudo-self-compatibility. This has impeded grass breeders in their efforts to produce highly homozygous lines, purged of their genetic load. In self-incompatible genotypes, the process of purging may be facilitated by the fact that, by self-pollination of a single plant, it is possible to produce a larger progeny pool to select from at each self-pollination cycle. Selfing is efficient in eliminating deleterious alleles whose effect on the phenotype are clearly visible and can be selected against, but less effective on mildly deleterious alleles (Boakes and Wang, 2005). As a result, while selfing helps to reduce a large part of the mutation load, it can also accelerate the fixation of deleterious alleles that are difficult to detect. Also, repeated selfing from a highly heterozygous genotype likely results in a reduction of genome size due to the purging of transposable elements and chromosomal knobs, as shown in *Z. mays* (Roessler *et al.*, 2019). Given the absence of empirical data on the impact of systematic selfing in forage grasses, it is difficult to envisage the impact of purging efforts and their effect on traits of agronomic importance. However, as recently demonstrated in potato (*Solanum tuberosum*), overcoming inbreeding depression by incorporating SC in selected germplasm is feasible and provides the possibility of creating novel, valuable inbred germplasm for *F*_1_ hybrid breeding (Lindhout *et al.*, 2011; Jansky *et al.*, 2016).

### Expanding the self-compatible germplasm pool for breeding

Forage grass breeders use a number of species, inter-species and inter-generic hybrids of the *Lolium–Festuca* complex but SC sources are currently only available in a restricted range of *L. perenne* genotypes and in its naturally self-compatible relatives, such as *L. temulentum* and flaxfield ryegrass (*Lolium remotum*). For effective exploitation of SC in breeding programmes it is crucial that a broad base of self-compatible germplasm is available. Introgressing SC within the *Lolium* genus would present little difficulty because all species have the same ploidy level (2*n* = 2*x* = 14) and show high levels of homoeologous chromosome pairing in interspecific hybrid offspring (Jauhar, 1975*a*). A straightforward approach of crossing two diploid individuals to produce, at least partially, fertile offspring followed by continued backcrossing could be utilized, as performed by Thorogood and Hayward (1992) and Yamada (2001) to transfer SC from *L. temulentum* to *L. perenne* and *L. multiflorum*. Autotetraploidy in *L. perenne* and *L. multiflorum*, induced by colchicine treatment of seeds or vegetative tillers, is exploited routinely in forage grass breeding programmes and transfer of SC into autotetraploid ryegrasses may be desirable. Although it was assumed, based on Stebbin’s seminal research, that polyploidy is inherently associated with SC (Stebbins, 1950), a relatively recent review showed no such relationship between the two traits (Mable, 2004). Certainly, polyploid *Festuca* species, as well as induced *L. perenne*, *L. multiflorum* and meadow fescue (*Festuca pratensis*) autopolyploids, exhibit an effective SI system and transfer of SC into a range of inter-fertile polyploid *Lolium* and *Festuca* species and hybrids could be proposed. For SC to be expressed in grass polyploids, introgressing SC into polyploid germplasm is a necessity and offers potential advantages over self-compatible diploid counterparts. For instance, theory predicts that polyploidy reduces the effect of the inbreeding depression that inevitably arises after repeated selfing of highly heterozygous plants (Lande and Schemske, 1985; Husband and Schemske, 1997). It has been shown that in autotetraploid (2*n* = 2*x* = 28) *L. perenne* the source of SC on LG 5 is functional (Do Canto *et al.*, 2020). Thus, it should be possible to produce self-compatible tetraploid varieties of *Lolium* species by first introgressing SC from diploid *L. perenne* and, after repeated backcrossing, inducing genome doubling in the self-compatible offspring through colchicine treatment (Morgan, 1976).

Species within the wider *Lolium–Festuca* (Section *Bovinae* = subg. *Schedonorus*) complex produce viable inter-generic hybrids (× *Festulolium*) that are of increasing interest to farmers because of their inherent superior abiotic stress tolerance and adaptability to a range of climate change scenarios (Humphreys *et al.*, 2014; Mäkinen *et al.*, 2018). Although there are clearly genetic and physical incongruities between the two genera in these hybrids, such as differences in gross chromosome morphology and size and, in many cases, chromosome number (ploidy level) that cause reduced hybrid fertility, there is a high degree of orthology and co-linearity (Jones *et al.*, 2002; Alm *et al.*, 2003). This offers the opportunity to introgress SC into a wider variety of agronomically important grass species. In addition, it leads to the prospect of producing lines having beneficial traits from both the *Lolium* and the *Festuca* genus. In fact, transferring useful traits in the *Lolium–Festuca* complex by repeated backcrossing of an interspecific hybrid with one of its parent species has found wide application in grass breeding. Introgression efforts have focused on backcrossing traits such as drought (Humphreys *et al.*, 2005) and freezing tolerance (Kosmala *et al.*, 2007), winter hardiness (Tamura *et al.*, 2017) and disease resistance (Płażek *et al.*, 2018) from the generally more robust *Festuca* species to the higher-yielding and nutritionally superior *Lolium* species (Kopecký *et al.*, 2017). For the successful transfer of SC sources across the complex, careful consideration must be given to both the degree of chromosome homology between the donor and recipient species along with differences in ploidy level, where diploid, triploid, tetraploid and hexaploid examples of the *Festuca* genus are found in nature. Transferring SC between *Lolium* and diploid meadow fescue (*F. pratensis*) is straightforward and natural hybrids are commonly found where the species are sympatric. Though previous reports have shown difficulties in producing seeds in sufficient quantity from the interspecific hybrids between *L. perenne* and *F. pratensis*, embryo rescue can increase the numbers of individuals recovered (Reusch, 1959; Kinoshita, 2007). Although hybrids are usually male sterile, further generations can be obtained through backcrossing to either of the parent species. Another approach used to alleviate male sterility is to artificially induce tetraploidy by treatment of the parent plants, or the *F*_1_ hybrid itself, with colchicine (Crowder, 1953; King *et al.*, 1998). Repeated rounds of backcrossing to diploid plants with SC selection would restore diploidy whilst retaining the source of SC. Alternatively, the induced tetraploid hybrids can be used to produce tetraploid populations. Following the demonstration of SC being expressed in autotetraploid *L. perenne* (Do Canto *et al.*, 2020), it has been shown that the SC source reported by Slatter *et al.* (2020) is readily expressed in *L. perenne* × *F. pratensis* tetraploids derived from crosses between self-compatible *L. perenne* and self-incompatible *F. pratensis*, generating viable seeds after selfing (D Thorogood, unpubl. data). Thus, the generic *Lolium* × *Festuca* hybrids, × *Festulolium*, provide a valuable target for transferring SC, especially as they display heterosis and reduced genetic load brought about by the combination of contrasting genomes (Comai, 2005). Consequently, the degree of inbreeding depression in polyploids would be reduced at each cycle of self-pollination, allowing the gradual removal of recessive mutations over successive generations more easily than in diploids. However, for full exploitation of fixed heterosis in self-compatible hybrid lines, the problem of genetic instability and reduced fertility due to partial homoeologous chromosome pairing, resulting in segmental allopolyploidy, needs to be resolved (Svačina *et al.*, 2020). The fact that many of the parental *Festuca* species are true allopolyploids with complete homeologous pairing indicates genetic control of chromosome pairing that can potentially be exploited (Jauhar, 1975*b*; Kopecký *et al.*, 2009, 2017; Svačina *et al.*, 2020). If successful, the ability to fix heterosis in self-compatible allopolyploid lines would negate the challenges of producing *F*_1_ hybrids.

### Immortalized populations for trait mapping

Trait-mapping projects in grass species are often based on *F*_1_ or *F*_2_ populations originating from a two-way pseudo-test cross between two highly heterozygous parents with contrasting traits. The substantial segregation shown in the progeny makes them suitable for mapping.

Such pseudo *F*_1_/*F*_2_ populations have been genotyped and phenotyped to identify QTL for several agronomical and physiological traits in *L. perenne* (Muylle *et al.*, 2005; Barre *et al.*, 2009), *L. multiflorum* (Studer *et al.*, 2007), *F. pratensis* (Ergon *et al.*, 2006; Alm *et al.*, 2011), tall fescue (*Festuca arundinacea*) (Saha *et al.*, 2009) and cock’s-foot (*Dactylis glomerata*) (Kallida *et al.*, 2016; Zhao *et al.*, 2016). Because single *F*_1_ plants cannot be selfed, pseudo-test crosses or full-sib matings of *F*_1_ plants have been widely employed to construct pseudo-backcross or pseudo-*F*_2_ populations. An example is the *L. perenne* VrnA population, a two-way pseudo-testcross *F*_2_ population initially constructed to identify QTL underlying vernalization response genes (Jensen *et al.*, 2005). In *L. multiflorum* and *F. pratensis*, several studies report the development of pseudo-*F*_2_ populations by intermating several full-sib *F*_1_ plants (Inoue *et al.*, 2004; Ergon *et al.*, 2013, 2016; Wang *et al.*, 2016). Only three ‘true’ *F*_2_ populations, all developed in *L. perenne*, are currently available among grasses: ‘*F*_2_ Biomass’, ‘WSC *F*_2_’ and ‘*F*_2_ IOWA’. Self-compatible plants produced by obligate selfing were used for their development, and thus it has been possible to create them by self-pollinating a single *F*_1_ individual. These populations have been employed in a handful of genetic mapping studies (Thorogood *et al.*, 2005; Turner *et al.*, 2006; Tomaszewski *et al.*, 2012; Arias-Aguirre *et al.*, 2013; Foito *et al.*, 2015; Do Canto *et al.*, 2018). Moreover, individuals of the *F*_2_ Biomass have served as starting material for the creation of the only immortalized population so far developed in forage grasses (Velmurugan *et al.*, 2018).

Though mapping efforts have had successful outcomes, SI and subsequent enforced heterozygosity means that most mapping populations in grasses consist of individuals that can only be maintained vegetatively. This is time-consuming and can compromise genetic integrity over time through somatic mutation and age-related genetic developmental changes or simply through human handling errors. The death of individual genotypes, causing a reduction in population size over time, will also reduce the size and genetic value of the mapping population. That is why the development of self-compatible germplasm resources for mapping important agronomic traits through the creation of inbred lines such as RILs and double haploids for broad-scale mapping (Hina *et al.*, 2020) and subsequently NILs for fine-mapping specific genomic regions and candidate gene identification (Zhang *et al.*, 2021) is potentially valuable. These germplasm resources present the advantage of enabling multiple independent mapping projects to analyse a single line, as they are easily stored and distributed as seeds along with being considered ‘immortal’ due to their homozygous nature and capacity to be maintained by self-pollination. A more systematic use of reference immortal populations will help not only in the identification of QTL underlying genes of interest, but also with developing molecular markers that can assist in trait selection.

### Dissection of heterosis

Heterosis is a complex trait expressed through the action of multiple loci (Schnable and Springer, 2013). In rice (*Oryza sativa*) (Hua *et al.*, 2002, 2003; Qu *et al.*, 2012; Zhou *et al.*, 2012; Zhen *et al.*, 2017) and *Z. mays* (Frascaroli *et al.*, 2007; Tang *et al.*, 2010; Giraud *et al.*, 2017; Wang *et al.*, 2016), genomics and QTL mapping approaches have allowed the identification of major loci associated with heterosis and have provided insights into its genetic architecture. Despite heterotic loci in grasses being unknown, heterotic levels have been reported for different types of experimental hybrids (Foster, 1971*a*, *b*, 1973; Posselt, 1993, 2010; Anhalt *et al.*, 2009; Barrett *et al.*, 2010; O’Connor *et al.*, 2015; Wang *et al.*, 2016), representing an untapped resource that breeders should capture. However, such unexplored potential can only be exploited with the identification of heterotic groups. In forage grasses, well-defined heterotic groups are not available (Vogt *et al.*, 2020). This is explained by the high relatedness of plant material within and between different breeding programmes, often the result of exchange of material among breeders or common initial germplasm. However, high-throughput genotyping approaches have proved successful in capturing the genetic diversity contained in germplasm and dividing it into different subpopulations (Vogt *et al.*, 2020). In this regard, the extensive use of homozygous self-compatible genotypes can help to diverge and fix the diverse gene pool contained in such different clusters, and to structure and define different heterotic groups. This will provide breeders with strategies to effectively select parental genotypes to be used for hybrid crosses and for the prediction of hybrid performance. In addition, the availability of well-diverged heterotic groups, coupled with advancement in sequencing technologies will allow the identification of genomic heterotic loci, for a better understanding of the genetic regulation of this complex phenomenon.

## CONCLUDING REMARKS

Identifying and characterizing the basis of SC in grasses serves to provide a better understanding of the genes involved in the induction of SC, and knowledge of how SC arises in normally self-incompatible species can help biologists determine the pathways involved in the SI process in grasses. This offers insights into the evolutionary relationship of the grass SI system with other SI systems and, more widely, with other cell–cell recognition processes in living organisms. The subsequent exploitation of SC has the potential to promote more effective breeding methodologies based on the development of population or *F*_1_ hybrids exploiting genetic variation for hybrid vigour. Fundamental studies of the genetic basis of the heterosis that SC facilitates will be critical for targeted practical application. The systematic investigation of the agronomic performance of hybrids and the design of efficient seed production systems will be pivotal for efficient production of first-generation hybrid or pure-line cultivars. Afforded by SC mutations, research exploring heterosis in normally outcrossing species, with high levels of accumulated heterozygosity and genetic diversity, offers the prospect of contributing significantly to our understanding of heterosis currently gained from model species and self-fertile crops.

## SUPPLEMENTARY DATA

Supplementary data are available online at https://academic.oup.com/aob and consist of the following. Table S1: summary of published literature on SI breakdown to SC in flowering plants. References S1: references cited in Table S1.

mcab043_suppl_Supplementary-Material-S01

mcab043_suppl_Supplementary-Material-S02
