# Supplementary material for: Characterization and practical use of self-compatibility in outcrossing grass species
Source: Ann Bot. 2021 Mar 23;127(7):841–52. doi: 10.1093/aob/mcab043 (PMC8225281; doi:10.1093/aob/mcab043)
Supplement: mcab043_suppl_Supplementary-Material-S01 [file mcab043_suppl_Supplementary-Material-S01.docx]

References for citations in supplementary Table 1.

**Ai Y, Kron E, Kao T**. **1991**. S-alleles are retained and expressed in a self-compatible cultivar of Petunia hybrida. *Molecular & General Genetics* **230**: 353–358.

**Arias-Aguirre A, Studer B, Canto JD, Frei U, Lübberstedt T**. **2013**. Mapping a New Source of Self-fertility in Perennial Ryegrass (*Lolium perenne* L.). *Plant Breeding and Biotechnology* **1**: 385–395.

**Atwood SS**. **1942**. Genetics of pseudo-self-compatibility and its relation to cross-incompatibility in *Trifolium repens*. *Journal of Agricultural Research* **64**: 699–700.

**Bachmann JA, Tedder A, Laenen B, Fracassetti M, Désamoré A, Lafon‐Placette C, Steige KA, Callot C, Marande W, Neuffer B, Bergès H, Köhler C, Castric V, Slotte T**. **2019**. Genetic basis and timing of a major mating system shift in Capsella. *New Phytologist* **224**: 505–517.

**Baldwin SJ, Schoen DJ**. **2017**. Genetic variation for pseudo-self-compatibility in self-incompatible populations of *Leavenworthia alabamica* (Brassicaceae). *New Phytologist* **213**: 430–439.

**Beppu K, Komatsu N, Yamane H, Yaegaki H, Yamaguchi M, Tao R, Kataoka I**. **2005**. *Se* -haplotype confers self-compatibility in Japanese plum ( *Prunus salicina* Lindl.). *The Journal of Horticultural Science and Biotechnology* **80**: 760–764.

**Bernatzky R, Miller D D**. **1994**. Self-incompatibility is codominant in intraspecific hybrids of self-compatible and self-incompatible *Lycopersicon peruvianum* and *L. hirsutum* based on protein and DNA marker analysis. *Sexual Plant Reproduction* **7**: 297–302.

**Bixby PJ, Levin DA**. **1996**. Response to selection on autogamy in *Phlox*. *Evolution* **50**: 892–899.

**Boggs NA, Nasrallah JB, Nasrallah ME**. **2009**. Independent 5-locus mutations caused self-fertility in *Arabidopsis thaliana*. *PLoS Genetics* **5**.

**Brewbaker JL, Natarajan AT**. **1960**. Centric Fragments and Pollen-Part Mutation of Incompatibility Alleles in *Petunia*. *Genetics* **45**: 699–704.

**Cachi AM, Wünsch A**. **2011**. Characterization and mapping of non-S gametophytic self-compatibility in sweet cherry (*Prunus avium* L.). *Journal of Experimental Botany* **62**: 1847–1856.

**Chantha S-C, Herman AC, Platts AE, Vekemans X, Schoen DJ**. **2013**. Secondary Evolution of a Self-Incompatibility Locus in the Brassicaceae Genus *Leavenworthia* (JB Nasrallah, Ed.). *PLoS Biology* **11**: e1001560.

**Clark KR, Okuley JJ, Collins PD, Sims TL**. **1990**. Sequence variability and developmental expression of S-alleles in self-incompatible and pseudo-self-compatible *Petunia*. *The Plant Cell* **2**: 815–826.

**Company RS i, Kodad O, Martí AF i, Alonso JM**. **2015**. Mutations conferring self-compatibility in *Prunus* species: From deletions and insertions to epigenetic alterations. *Scientia Horticulturae* **192**: 125–131.

**Do Canto J, Studer B, Frei U, Lübberstedt T**. **2018**. Fine mapping a self-fertility locus in perennial ryegrass. *Theoretical and Applied Genetics* **131**: 817–827.

**Dwyer KG, Berger MT, Ahmed R, Hritzo MK, McCulloch AA, Price MJ, Serniak NJ, Walsh LT, Nasrallah JB, Nasrallah ME**. **2013**. Molecular characterization and evolution of self-incompatibility genes in *Arabidopsis thaliana*: the case of the Sc haplotype. *Genetics* **193**: 985–994.

**East EM**. **1932**. Studies on Self-Sterility. IX. the Behavior of Crosses between Self-Sterile and Self-Fertile Plants. *Genetics* **17**: 175–202.

**Flaschenriem DR, Ascher PD**. **1979**. S allele discrimination in styles of *Petunia hybrida* bearing stylar-conditioned pseudo-self-compatibility. *Theoretical and Applied Genetics* **55**: 23–28.

**Fujimoto R, Sugimura T, Fukai E, Nishio T**. **2006**. Suppression of gene expression of a recessive SP11/SCR allele by an untranscribed SP11/SCR allele in *Brassica* self-incompatibility. *Plant Molecular Biology* **61**: 577–587.

**García-Valencia LE, Bravo-Alberto CE, Wu H-M, Rodríguez-Sotres R, Cheung AY, Cruz-García F**. **2017**. SIPP, a Novel Mitochondrial Phosphate Carrier, Mediates in Self-Incompatibility. *Plant Physiology* **175**: 1105–1120.

**Gaude T, Friry A, Heizmann P, Mariac C, Rougier M, Fobis I, Dumas C**. **1993**. Expression of a self-incompatibility gene in a self-compatible line of *Brassica oleracea*. *The Plant Cell* **5**: 75–86.

**Golz JF, Clarke AE, Newbigin E, Anderson M**. **1998**. A relic S-RNase is expressed in the styles of self-compatible *Nicotiana sylvestris*. *The Plant Journal* **16**: 591–599.

**Golz JF, Oh H-Y, Su V, Kusaba M, Newbigin E**. **2001**. Genetic analysis of *Nicotiana* pollen-part mutants is consistent with the presence of an S-ribonuclease inhibitor at the S locus. *Proceedings of the National Academy of Sciences* **98**: 15372–15376.

**Goring DR, Glavin TL, Schafer U, Rothstein SJ**. **1993**. An S receptor kinase gene in self-compatible *Brassica napus* has a 1-bp deletion. *The Plant Cell* **5**: 531–539.

**Hauck NR, Ikeda K, Tao R, Iezzoni AF**. **2006**. The mutated S1-haplotype in sour cherry has an altered S-haplotype-specific F-box protein gene. *Journal of Heredity* **97**: 514–520.

**Hayman DL, Richter J**. **1992**. Mutations affecting self-incompatibility in *Phalaris coerulescens* *desf*. (Poaceae). *Heredity* **68**: 495–503.

**Hee-Jeong J, Nasar Uddin A, Jong-In P, Senthil Kumar T, Hye-Ran K, Yong-Gu C, Ill-Sup N**. **2014**. Analysis of S-locus and expression of S-alleles of self-compatible rapid-cycling *Brassica oleracea* “TO1000DH3.” *Molecular Biology Reports* **41**: 6441–6448.

**Henny RJ, Ascher PD**. **1976**. The inheritance of pseudo-self-compatibility (PSC) in *Nemesia strumosa* Benth. *Theoretical and Applied Genetics* **48**: 185–195.

**Hogenboom NG**. **1972**. Breaking breeding barriers in Lycopersicon. 3. Inheritance of self-compatibility in *L. peruvianum* (L.) Mill. *Euphytica* **21**: 244–256.

**Hosaka K, Hanneman RE**. **1998**. Genetics of self-compatibility in a self-incompatible wild diploid potato species *Solanum chacoense*. 2. Localization of an S locus inhibitor (Sli) gene on the potato genome using DNA markers. *Euphytica* **103**: 265–271.

**Indriolo E, Tharmapalan P, Wright SI, Goring DR**. **2012**. The ARC1 E3 ligase gene is frequently deleted in self-compatible Brassicaceae species and has a conserved role in *Arabidopsis lyrata* self-pollen rejection. *The Plant Cell* **24**: 4607–4620.

**Jung H-J, Park J-I, Ahmed N, Chung M-Y, Kim H-R, Yongbo D, Lee S-S, Nou I-S**. **2014**. Characterization of self-incompatibility genes in the intergeneric hybrid *xBrassicoraphanus*. *Plant Systematics and Evolution* **300**: 1903–1911.

**Kondo K, Yamamoto M, Matton DP, Sato T, Hirai M, Norioka S, Hattori T, Kowyama Y**. **2002**. Cultivated tomato has defects in both *S‐RNase* and *HT* genes required for stylar function of self‐incompatibility. *The Plant Journal* **29**: 627–636.

**Kowyama Y, Kunz C, Lewis I, Newbigin E, Clarke AE, Anderson MA**. **1994**. Self-compatibility in a *Lycopersicon peruvianum* variant (LA2157) is associated with a lack of style S-RNase activity. *Theoretical and Applied Genetics* **88**: 859–864.

**Li W, Yang Q, Gu Z, Wu C, Meng D, Yu J, Chen Q, Li Y, Yuan H, Wang D, Li T**. **2016**. Molecular and genetic characterization of a self-compatible apple cultivar, ‘CAU-1’. *Plant Science* **252**: 162–175.

**Litzow ME, Ascher PD**. **1983**. The inheritance of pseudo-self compatibility (PSC) in *Raphanus sativus* L. *Euphytica* **32**: 9–15.

**Liu W, Pei M, Zhang A**. **2018**. Studying on the strictly self-compatibility mechanism of ‘Liuyefeitao’ peach (*Prunus persica* L.). *PLoS ONE* **13**: 1–17.

**Liu P, Sherman-Broyles S, Nasrallah ME, Nasrallah JB**. **2007**. A cryptic modifier causing transient self-incompatibility in *Arabidopsis thaliana*. *Current biology : CB* **17**: 734–740.

**Lundqvist A**. **1958**. Self-Incompatibility in Rye: IV. Factors related to self‐seeding. *Hereditas* **44**: 193–256.

**Lundqvist A**. **1968**. The Mode of Origin of Self Fertility in Grasses. *Hereditas* **59**: 413–426.

**Mable BK, Hagmann J, Kim S-T, Adam A, Kilbride E, Weigel D, Stift M**. **2017**. What causes mating system shifts in plants? *Arabidopsis lyrata* as a case study. *Heredity* **118**: 52–63.

**Mable BK, Robertson AV, Dart S, Di Berardo C, Witham L**. **2005**. Breakdown of self-incompatibility in the perennial *Arabidopsis lyrata* (Brassicaceae) and its genetic consequences. *Evolution; International Journal of Organic Evolution* **59**: 1437–1448.

**Manzanares C, Barth S, Thorogood D, Byrne SL, Yates S, Czaban A, Asp T, Yang B, Studer B**. **2016**. A gene encoding a DUF247 domain protein cosegregates with the S self-incompatibility locus in perennial ryegrass. *Molecular Biology and Evolution* **33**: 870–884.

**Marchese A, Bošković RI, Caruso T, Raimondo A, Cutuli M, Tobutt KR**. **2007**. A new self-compatibility haplotype in the sweet cherry ‘Kronio’, S5′, attributable to a pollen-part mutation in the SFB gene. *Journal of Experimental Botany* **58**: 4347–4356.

**Markova DN, Petersen JJ, Yam SE, Corral A, Valle MJ, Li W, Chetelat RT**. **2017**. Evolutionary history of two pollen self-incompatibility factors reveals alternate routes to self-compatibility within *Solanum*. *American Journal of Botany* **104**: 1904–1919.

**Martin FW**. **1963**. Distribution and Interrelationships of Incompatibility Barriers in the *Lycopersicon hirsutum* Humb. and Bonpl. Complex. *Evolution* **17**: 519–528.

**Mather K**. **1943**. Specific differences in *Petunia*. *Journal of Genetics* **45**: 215–235.

**Muñoz-Sanz JV, Zuriaga E, Badenes ML, Romero C**. **2017**. A disulfide bond A-like oxidoreductase is a strong candidate gene for self-incompatibility in apricot (*Prunus armeniaca*) pollen. *Journal of Experimental Botany* **68**: 5069–5078.

**Nasrallah ME**. **1974**. Genetic control of quantitative variation in self-incompatibility proteins detected by immunodiffusion. *Genetics* **76**: 45–50.

**Nasrallah ME, Kandasamy MK, Nasrallah JB**. **1992**. A genetically defined trans-acting locus regulates S-locus function in *Brassica*. *The Plant Journal* **2**: 497–506.

**Nasrallah ME, Liu P, Nasrallah JB**. **2002**. Generation of self-incompatible *Arabidopsis thaliana* by transfer of two S locus genes from A. lyrata. *Science (New York, N.Y.)* **297**: 247–249.

**Nasrallah ME, Liu P, Sherman-Broyles S, Boggs NA, Nasrallah JB**. **2004**. Natural variation in expression of self-incompatibility in *Arabidopsis thaliana*: Implications for the evolution of selfing. *Proceedings of the National Academy of Sciences* **101**: 16070–16074.

**Nasrallah JB, Liu P, Sherman-Broyles S, Schmidt R, Nasrallah ME**. **2007**. Epigenetic mechanisms for breakdown of self-incompatibility in interspecific hybrids. *Genetics* **175**: 1965–1973.

**Nasrallah JB, Rundle SJ, Nasrallah ME**. **1994**. Genetic evidence for the requirement of the *Brassica* S-locus receptor kinase gene in the self-incompatibility response. *The Plant Journal* **5**: 373–384.

**Nasrallah ME, Wallace DH**. **1968**. The influence of modifier genes on the intensity and stability of self-incompatibility in cabbage. *Euphytica* **17**: 495–503.

**Okamoto S, Odashima M, Fujimoto R, Sato Y, Kitashiba H, Nishio T**. **2007**. Self-compatibility in *Brassica napus* is caused by independent mutations in S-locus genes. *The Plant Journal* **50**: 391–400.

**Oliveira MM**. **2001**. Molecular identification of *S*-genotypes of almond (*Prunus dulcis*). *Acta Horticulturae*: 575–580.

**Ono K, Akagi T, Morimoto T, Wónsch A, Tao R**. **2018**. Genome re-sequencing of diverse sweet cherry (*Prunus avium*) individuals reveals a modifier gene mutation conferring pollen-part self-compatibility. *Plant and Cell Physiology* **59**: 1265–1275.

**Pandey KK**. **1965**. Centric Chromosome Fragments and Pollen-Part Mutation of the Incompatibility Gene in *Nicotiana alata*. *Nature* **206**: 792–795.

**Robacker CD, Ascher PD**. **1982**. Effect of selection for pseudo-self compatibility in advanced inbred generations of *Nemesia strumosa* Benth. *Euphytica* **31**: 591–601.

**Royo J, Kunz C, Kowyama Y, Anderson M, Clarke AE, Newbigin E**. **1994**. Loss of a histidine residue at the active site of S-locus ribonuclease is associated with self-compatibility in *Lycopersicon peruvianum*. *Proceedings of the National Academy of Sciences of the United States of America* **91**: 6511–6514.

**Sassa H, Hirano H, Nishio T, Koba T**. **1997**. Style-specific self-compatible mutation caused by deletion of the S-RNase gene in Japanese pear (*Pyrus serotina*). *The Plant Journal* **12**: 223–227.

**Sharma JR, Murty BR**. **1979**. Changes in genetic background under selection influencing the expression of self-incompatibility in *Brassica campestris* var. brown sarson. *Genetica* **51**: 45–53.

**Slatter LM, Barth S, Manzanares C, Velmurugan J, Place I, Thorogood D**. **2020**. A new genetic locus for self-compatibility in the outcrossing grass species perennial ryegrass (*Lolium perenne*). *Annals of Botany*: mcaa140.

**Sonneveld T, Tobutt KR, Vaughan SP, Robbins TP**. **2005**. Loss of Pollen-S Function in Two Self-Compatible Selections of *Prunus avium* Is Associated with Deletion/Mutation of an S Haplotype-Specific F-Box Gene. *The Plant Cell* **17**: 37–51.

**Takahashi H**. **1973**. Genetical and physiological analysis of pseudo- self-compatibility in *Petunia hybrida*. *The Japanese journal of genetics* **48**: 27–33.

**Tao R, Watari A, Hanada T, Habu T, Yaegaki H, Yamaguchi M, Yamane H**. **2007**. Self-compatible peach (*Prunus persica*) has mutant versions of the S haplotypes found in self-incompatible *Prunus species*. *Plant molecular biology* **63**: 109–23.

**Thompson KF, Taylor JP**. **1966**. The breakdown of self-incompatibility in cultivars of *Brassica oleracea*. *Heredity* **21**: 637–648.

**Thompson KF, Taylor JP**. **1971**. Self-compatibility in kale. *Heredity* **27**: 459–471.

**Thorogood D, Armstead IP, Turner LB, Humphreys MO, Hayward MD**. **2005**. Identification and mode of action of self-compatibility loci in *Lolium perenne* L. *Heredity* **94**: 356–63.

**Thorogood D, Hayward MD**. **1992**. Self-compatibility in *Lolium temulentum* L: its genetic control and transfer into L. perenne L. and L. multiflorum Lam. *Heredity* **68**: 71–78.

**Tochigi T, Udagawa H, Li F, Kitashiba H, Nishio T**. **2011**. The self-compatibility mechanism in *Brassica napus* L. is applicable to F1 hybrid breeding. *Theoretical and Applied Genetics* **123**: 475–482.

**Townsend CE**. **1966a**. Self-Compatibility Studies with Diploid Alsike Clover, *Trifolium hybridum* L. II. Inheritance of a Self-Compatibility Factor with Gametophytic and Sporophytic Characteristics. *Crop Science* **6**: 415–419.

**Townsend CE**. **1966b**. Self-Compatibility Response to Temperature and the Inheritance of the Response in Tetraploid Alsike Clover, Trifolium hybridum L.1. *Crop Science* **6**: 409–414.

**Tsuchimatsu T, Suwabe K, Shimizu-Inatsugi R, Isokawa S, Pavlidis P, Städler T, Suzuki G, Takayama S, Watanabe M, Shimizu KK**. **2010**. Evolution of self-compatibility in *Arabidopsi*s by a mutation in the male specificity gene. *Nature* **464**: 1342–1346.

**Tsukamoto T, Ando T, Kokubun H, Watanabe H, Masada M, Zhu X, Marchesi E, Kao TH**. **1999**. Breakdown of self-incompatibility in a natural population of *Petunia axillaris* (Solanaceae) in Uruguay containing both self-incompatible and self-compatible plants. *Sexual Plant Reproduction* **12**: 6–13.

**Tsukamoto T, Ando T, Takahashi K, Omori T, Watanabe H, Kokubun H, Marchesi E, Kao T**. **2003**. Breakdown of self-incompatibility in a natural population of *Petunia axillaris* caused by loss of pollen function. *Plant Physiology* **131**: 1903–1912.

**Ushijima K, Yamane H, Watari A, Kakehi E, Ikeda K, Hauck NR, Iezzoni AF, Tao R**. **2004**. The S haplotype-specific F-box protein gene, *SFB*, is defective in self-compatible haplotypes of *Prunus avium* and *P. mume*. *The Plant Journal* **39**: 573–586.

**Vilanova S, Badenes ML, Burgos L, Martínez-Calvo J, Llácer G, Romero C**. **2006**. Self-Compatibility of Two Apricot Selections Is Associated with Two Pollen-Part Mutations of Different Nature. *Plant Physiology* **142**: 629–641.

**Voylokov AV, Fuong FT, Smirnov VG**. **1993**. Genetic studies of self-fertility in rye (*Secale cereale* L.). 1. The identification of genotypes of self-fertile lines for the Sf alleles of self-incompatibility genes. *Theoretical and Applied Genetics* **87**: 616–618.

**Watari A, Hanada T, Yamane H, Esumi T, Tao R, Yaegaki H, Yamaguchi M, Beppu K, Kataoka I**. **2007**. A Low Transcriptional Level of Se-RNase in the Se -haplotype Confers Self-compatibility in Japanese Plum. *Journal of the American Society for Horticultural Science* **132**: 396–406.

**Williams RD, Williams W**. **1947**. Genetics of red clover (*Trifolium pratense* L.); the frequency of incompatibility S alleles in two non-pedigree populations of red clover. *Journal of Genetics* **48**: 69–79.

**Wunsch A, Hormaza J**. **2004**. Genetic and molecular analysis in Cristobalina sweet cherry, a spontaneous self-compatible mutant. *Sexual Plant Reproduction* **17**: 203–210.

**Xiao Z, Han F, Hu Y, Xue Y, Fang Z, Yang L, Zhang Y, Liu Y, Li Z, Wang Y, Zhuang M, Lv H**. **2019**. Overcoming Cabbage Crossing Incompatibility by the Development and Application of Self-Compatibility-QTL- Specific Markers and Genome-Wide Background Analysis. *Frontiers in Plant Science* **10**.

**Xue Y, Zhang Y, Yang Q, Li Q, Cheng Z, Dickinson HG**. **2009**. Genetic features of a pollen-part mutation suggest an inhibitory role for the *Antirrhinum* pollen self-incompatibility determinant. *Plant Molecular Biology* **70**: 499–509.

**Yamane H, Ikeda K, Hauck NR, Iezzoni AF, Tao R**. **2003**. Self‐incompatibility (S) locus region of the mutated S6‐haplotype of sour cherry (*Prunus cerasus*) contains a functional pollen S allele and a non‐functional pistil S allele. *Journal of Experimental Botany* **54**: 2431–2437.

**Zhai W, Zhang J, Yang Y, Ma C, Liu Z, Gao C, Zhou G, Tu J, Shen J, Fu T**. **2014**. Gene expression and genetic analysis reveal diverse causes of recessive self-compatibility in *Brassica napus* L. *BMC Genomics* **15**: 1037.

**Zuriaga E, Muñoz-Sanz JV, Molina L, Gisbert AD, Badenes ML, Romero C**. **2013**. An S-Locus Independent Pollen Factor Confers Self-Compatibility in ‘Katy’ Apricot. *PLoS ONE* **8**.
